# Decoding Insulin Secretory Granule Maturation Using Genetically Encoded pH Sensors

**DOI:** 10.1021/acssensors.4c01885

**Published:** 2024-11-06

**Authors:** Wen Lin, Kaylee Tseng, Scott E. Fraser, Jason Junge, Kate L. White

**Affiliations:** 1 Department of Chemistry, Bridge Institute, USC Michelson Center for Convergent Bioscience, University of Southern California, Los Angeles, California 90089, United States; 2 Department of Biological Sciences, Bridge Institute, USC Michelson Center for Convergent Bioscience, Translational Imaging Center, University of Southern California, 1002 Childs Way, Los Angeles, California 90089, United States

**Keywords:** FLIM-FRET, genetically encoded, pancreatic beta cell, insulin secretory granule, maturation pathway, pH sensor

## Abstract

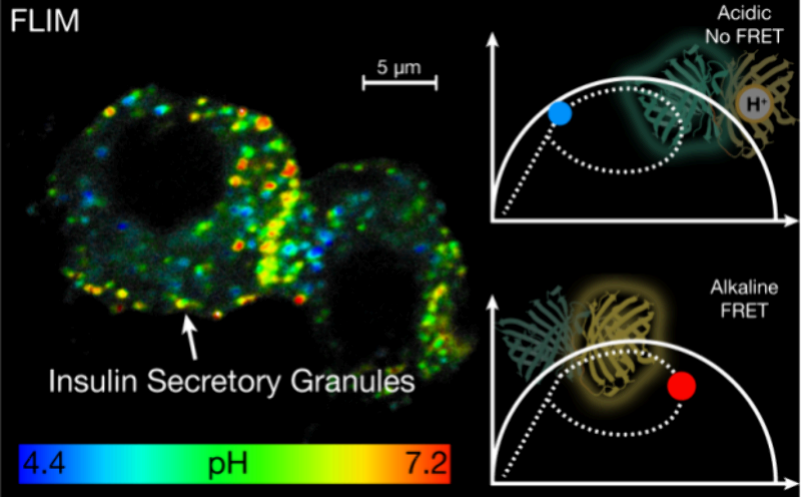

Insulin is a peptide hormone secreted from pancreatic beta cells to regulate blood glucose homeostasis. Maturation of active insulin occurs within insulin secretory granules (ISG) by acidification of the lumen and enzymatic cleavage of insulin before secretion. This process is dysregulated in diabetes, and many questions remain on how the cell controls insulin maturation. We address this gap in knowledge by designing two genetically encoded fluorescence pH sensors and a fluorescence lifetime imaging and analysis pipeline to monitor the pH of individual secretory ISGs within live cells at higher resolution and precision than previously possible. We observed different subpopulations of ISGs based on their pH and subcellular localization. Signals regulating metabolism vs membrane depolarization mobilize different subpopulations of ISGs for secretion, and we confirm that maturation signals acidify ISGs. We conclude that different signaling networks uniquely impact ISG mobilization and secretion. Future applications of these tools will be useful for exploring how these processes are dysregulated in diabetes and provide new paths for developing more effective treatments.

Insulin is secreted from pancreatic beta cells to regulate blood glucose homeostasis; however, this process is dysregulated in diabetes.^1^ Insulin is processed and stored in insulin secretory granules (ISGs) that emerge from the trans-Golgi network. ISG maturation is required for the secretion of active insulin. As the ISG matures, the ISG lumen acidifies, facilitating the enzymatic conversion of proinsulin to active insulin.^2^ ISG acidity is a common indicator of maturity.^3,4^ A critical gap in knowledge is understanding how the cell regulates ISG maturation. A better understanding of the protein signaling pathways that influence ISG pH will provide new mechanistic insights into insulin maturation and may offer new directions for therapeutic interventions.

With the advent of genetically encoded Förster resonance energy transfer (FRET) pH sensors,^5,6^ it is possible to monitor intracellular pH in living cells over time. However, achieving precise pH measurements from single ISGs is challenging because of their small size and fast movement (up to 1 μm/s).^7−9^ To achieve the resolution required to observe the pH dynamics of individual ISGs, we opted to pair FRET sensors with fluorescence lifetime imaging microscopy (FLIM).

FLIM-FRET is an approach to quantitative imaging that is growing in popularity due to its capability to precisely measure FRET efficiency without error-prone intensity-based calculations.^10^ With FLIM-FRET, FRET efficiency is measured through inversely proportional fluorescence lifetime (Figure 1A). Because FLIM is relatively insensitive to intensity readings, FLIM-FRET measurements are less impacted by discrepancies in sensor expression levels within and among cells, which makes it suitable for in cellulo measurements. To further improve the FLIM-FRET-based pH measurement, we employed phasor analysis^11^ with a complex wavelet filter^12^ instead of using conventional FLIM measurement where empirical lifetime is calibrated against pH (see Figure S1 for the full description of phasor analysis). Developed by Gratton et al. and widely adopted in the FLIM community, phasor analysis enables graphical representation of the lifetime distribution and is a powerful tool for easily separating different lifetime populations.^13,14^ Applying the complex wavelet filter^12^ to phasor analysis delivers precise measurement under low photon count conditions enabling speedy acquisition. Furthermore, this technique requires only single-channel acquisition, thereby reducing phototoxicity and improving the viability of live samples.^15^ Here, we optimize and validate genetically encoded FLIM-FRET pH sensors designed specifically for ISG pH range, enabling pH measurement at single ISG resolution.

**Figure 1:**
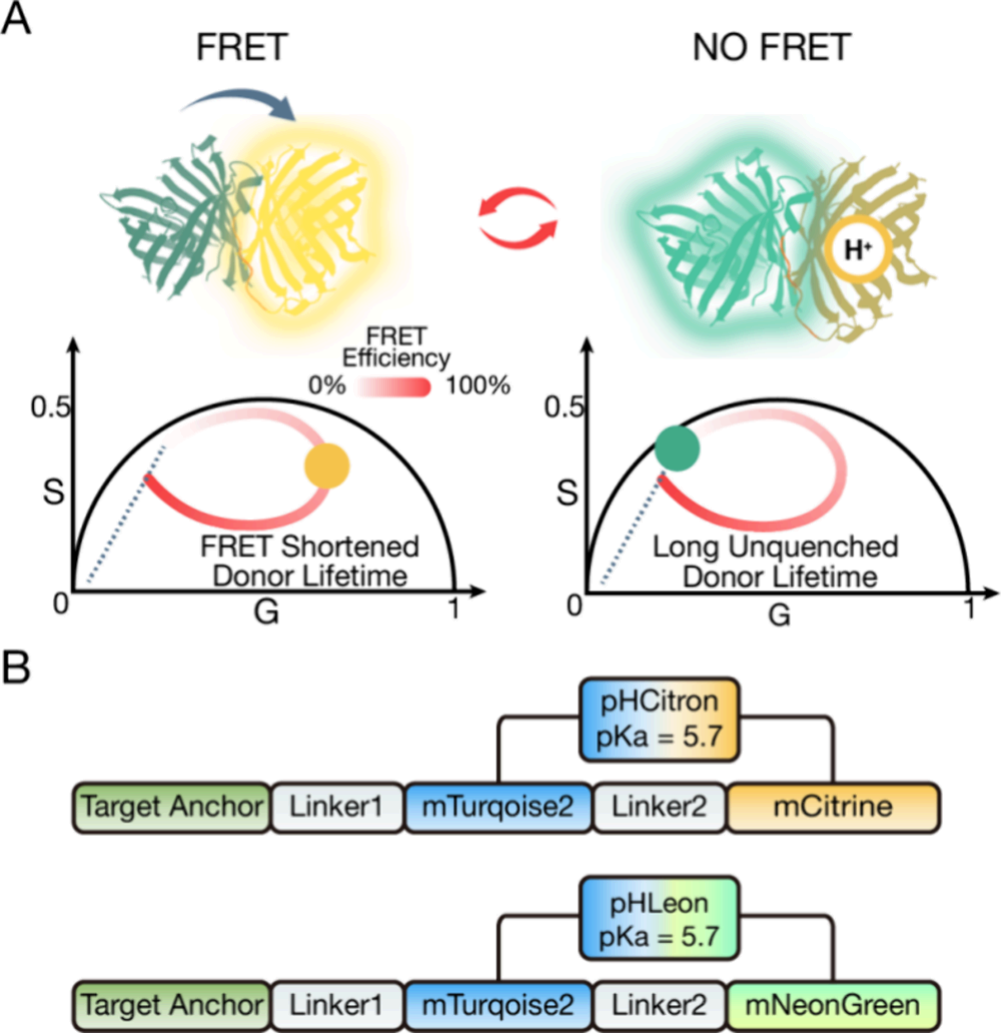
Design of the pH sensors. (A) Schematic of the molecular FRET mechanism of the pH sensors. Protonation deactivates the acceptor and disrupts FRET, resulting in a longer fluorescence lifetime than the FRET state, as shown on the phasor plots. (B) Sequence schematic of the two pH sensors used in this study.

## Experimental Methods

### Cell Culture and Transfection

INS-1E (rat insulinoma cell line) cells were generously provided by Dr. Pierre Maechler from the University of Geneva, Switzerland.^16^ The cells were cultured in supplemented RPMI 1640 medium at 37 °C with humidified air containing 5% carbon dioxide. INS-1E cells were passed once per week.

HEK293T cells were purchased from Invitrogen and cultured in DMEM at 37 °C with humidified air containing 10% carbon dioxide. HEK293T cells were passed twice per week.

For imaging experiments, the cells were plated on chambered cover glass slides (62407-0560, VWR) or polymer chambered coverslips (80806, ibidi GmbH) and were initially seeded at a density of 40,000 cells/cm^2^. The cells grew on these slides for 48 h to ensure adherence and confluency before transfection. Lipofectamine 3000 reagents (L3000001, Thermo Fisher Scientific) were used for transfection as per the instructions provided by the manufacturer.

### Sensor Construction

To enable expression in both mammalian and bacterial systems, two modular constructs containing mTurquoise2-mCitrine were synthesized by GeneScript. These constructs were created using the PCDNA3.1 and Pet28b backbones with the linker between mTurquoise2 and mCitrine adapted from the pH-Lemon construct.^17^

To achieve the ISG-targeted expression in INS-1E cells, neuropeptide Y (NPY) with a linker was subcloned into the N-terminus of mTurquoise2. Mitochondrial targeting was achieved by inserting the hsADCK3^18^ sequence into the N-terminus of mTurquoise2.

To create pHLeon constructs, mCitrine was replaced with mNeonGreen. The original construct of mNeonGreen was obtained from the Addgene repository (Addgene ID: 125139). The sequences of all the constructs mentioned above and their respective restriction sites are listed in the Supporting Information.

### In Cellulo Calibration

HEK293T adherent cells reaching 60,000 cells/cm^2^ confluency were transfected with PCDNA3.1 cytosolic sensor constructs. After 24 h, the cells were permeabilized in pH 7.4 calibration buffer with valinomycin and nigericin (P35379, Thermo Fisher Scientific) or 30 min before FLIM imaging. The field of view (FOV) containing approximately 15 cells was FLIM imaged as buffer pH was adjusted by adding citric acid. The amount of citric acid added and the resulting buffer pH were determined by a separate titration experiment (pH range: 4.0–7.6).

### Microscopy

Unless otherwise noted, all imaging experiments were performed on a Leica SP8 DIVE FALCON with 63*x*/1.4NA oil immersion objective or 63*x*/1.2NA water immersion objective. FLIM images were obtained via time-correlated single photon counting (TCSPC) through which photons emitted from the donor were collected between 450 and 500 nm. The excitation light source was an ultrafast Spectra-Physics InSight X3 tunable IR laser at 0.2 mW average power set at 870 nm for 2P excitation, pulsing at 80 MHz and 1 ps pulse width. For live cell imaging, each *z*-plane consisted of five to seven image repetitions, integrated until the median photon counts reached 50. For calibration experiments, a single *z*-plane was collected for protein solutions and permeated cell samples with 20 repetitions to ensure calibration accuracy. Single-cell images were acquired at a resolution of 512 × 512 pixels with a pixel size of approximately 60 nm. FOV captured a single cell, and the *z*-stack varied based on the cell’s height with a consistent *z*-step size of 300 nm.

### Stimulation Experiments

INS-1E cells were incubated for 30 min in a glucose-free Krebs-Ringer bicarbonate HEPES buffer (KRBH) before stimulation experiments, as commonly done for INS-1E secretion studies.^16^ Before introducing any stimuli, a set of cells (∼3 to 5) were identified and imaged with their coordinates recorded. Stimuli solutions were prepared at twice the concentration in KRBH buffer and were directly added to the starved cell samples during stimulation without aspiration to minimize cell disturbance. The same set of cells selected were then imaged and time-stamped to monitor their changes.

### Data Analysis and Availability

Collected image is subjected to segmentation for ISGs with Imaris 10 (Oxford Instruments) and cell boundary. Please refer to supplementary text for detailed data processing pipeline.

An approximate analysis workflow is summarized in a supplementary schematic (Figure S2).

## Results and Discussion

### Design and Validation of Genetically Encoded pH Indicator

To better accommodate the reported pH range of ISGs (pH 5–6), we modified the pH-Lemon^17^ sensor by replacing the acceptor EYFP (p*K*_a_ = 7.1) with mCitrine (p*K*_a_ = 5.7) and mNeonGreen (p*K*_a_ = 5.7). These new constructs were named pHCitron (pH-Lemon with mCitrine) and pHLeon (pH-Lemon with mNeonGreen) (Figure 1B).

To target these sensors to the ISG lumen, we fused NPY, a known luminal ISG marker,^19−22^ to the N-termini of the sensors and introduced them into cells via transient expression. We chose NPY to target our sensor rather than fusing the biosensor directly with insulin so we would not interfere with the natural enzyme processing and biochemical maturation of the peptide hormone. NPY-pHLeon and insulin showed colocalization, confirming that most of the NPY-targeted sensors are within ISGs (Figure S3).

To evaluate the sensors’ compatibility for monitoring ISG pH, we imaged INS-1E cells expressing NPY-pHCitron, NPY-pHLeon, and NPY-(pH-Lemon) using FLIM with phasor analysis. We also included the NPY-mTurquoise2 (donor-only) construct as a benchmark for the FLIM-FRET analysis. By comparing ISG lifetimes (Figure S4), we found that lowering the p*K*_a_ of the acceptor enhanced the lifetime dynamic range in ISGs, with pHLeon showing the greatest extension due to its higher native FRET ratio in the tandem construct.^23^ Our improvements in adjusting p*K*_a_ and the maximum FRET efficiency of the construct increased precision for ISG pH measurements.

Comparing the same data set analyzed in lifetime and phasor, phasor analysis showed better separation of distinct ISGs by their lifetime than lifetime fitting (Figure S5). We attribute this improvement to the compatibility of phasor analysis and the distinct mechanism of the FLIM-FRET pH sensors.^13^ Phasor analysis is especially advantageous in analyzing a linear combination of lifetime-distinct fluorophores. The sensor exists in an on- or off-state that is purely dependent on the protonation state of the acceptor chromophore (Figure 1A). In the on-state, the acceptor is deprotonated, and the sensor exhibits maximum FRET efficiency of the construct with a reduced lifetime. In the off-state, the acceptor is protonated, and the sensor exhibits minimal FRET efficiency with a longer lifetime. pH of the environment and p*K*_a_ of the chromophore dictate the proportion of these two states. This proportion can be intuitively measured with phasor analysis. This direct extrapolation of pH from the phasor and the higher dynamic range provided by these two sensors allowed photon-efficient and precise pH measurement. Eliminating empirical lifetime fitting and calibration based on lifetime consolidates measurement uncertainty. While highly optimized for TCSPC instruments, this technique applies to other FLIM imaging modalities equipped with phasor analysis.

### In Cellulo Calibration

**Figure 2:**
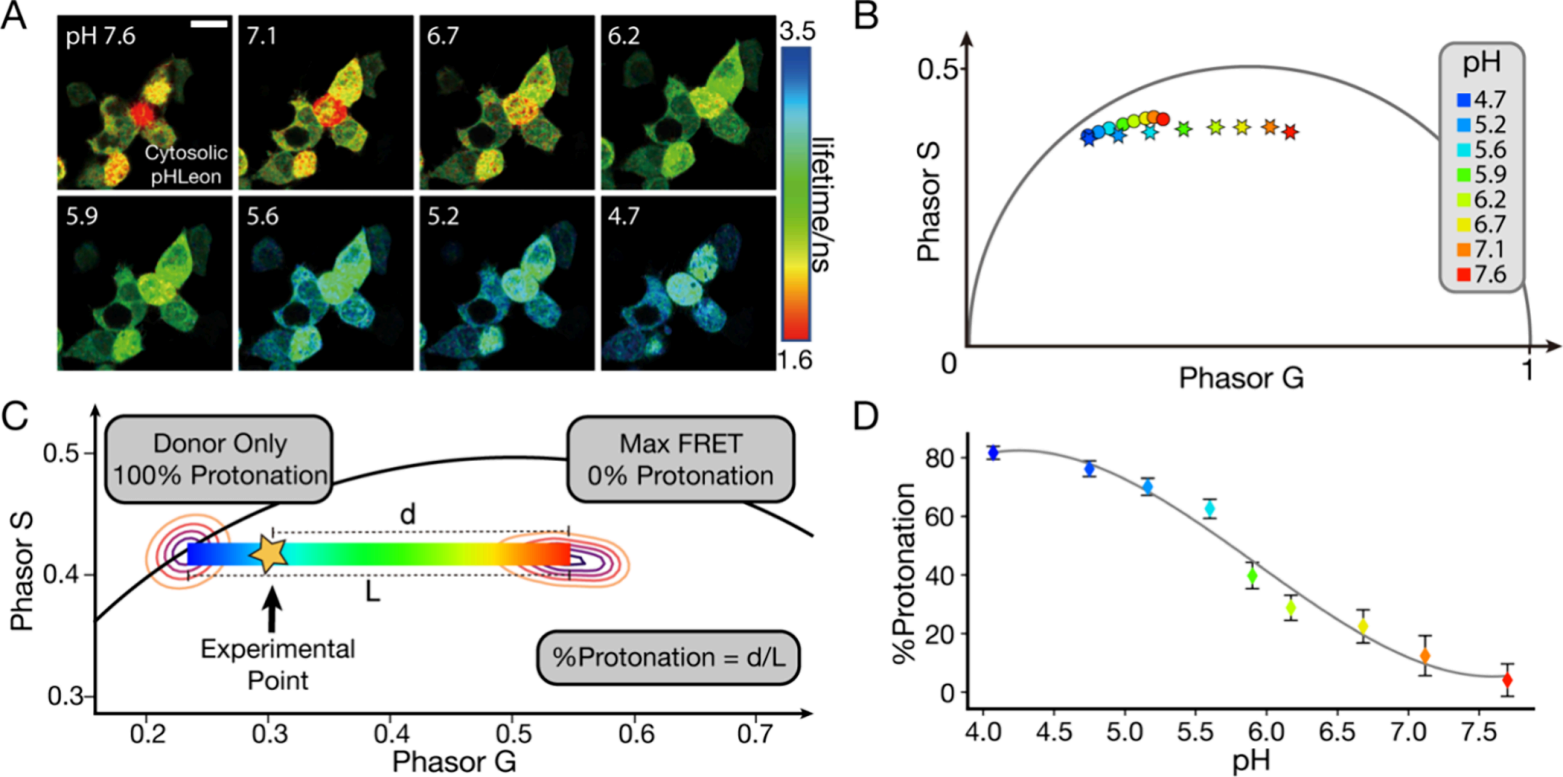
Calibration of the pH sensors. (A) pHLeon expressed in the cytoplasm of HEK293T cells in phosphate buffer (pH 7.6) titrated with citric acid. Pseudocolored lifetime FLIM images monitoring the same set of permeabilized cells at different pH are shown. Scale bar = 10 μm. (B) Simplified phasor histogram of in cellulo calibration of pHCitron (in dots) and pHLeon (in stars), indicating that pHLeon has a broader dynamic range. See Figure S1 for more details on phasor analysis (C) Use of phasor histogram to derive %Protonation, which relates to pH. The rainbow color bar indicates the calculated %Protonation and is used as a pseudocolor scheme in later figures. (D) Calibration curve of %Protonation vs pH, which is used to calculate pH in cellular experiments. The gray line is the third-order fit of data, and error bars denote the 50% interquartile range (IQR) of the phasor histogram to better represent experimental phasor data.

To validate the newly designed sensors and account for autofluorescence from cells, we conducted calibration of pH in cells. Our reliance on phasor analysis necessitates this step since the autofluorescence affects phasor lifetime readings in the channel where sensor lifetime is collected. We assume that autofluorescence characteristics are similar among mammalian cells. We expressed the sensor in the cytoplasm of HEK293T cells. These cells are easy to transfect and permeabilize, making them ideal for establishing an in cellulo calibration. On the same permeabilized cells, we see an expected change in phasor signal as the pH changes (Figure 2A,B), demonstrating the sensors’ ability to report environmental pH changes in a cellular environment.

To better represent the phasor signals, we implemented a metric called %Protonation calculated from the phasor coordinates (g, s) (Figure 2C). This quantifies the proportion of protonated sensors, which is directly related to pH. According to the principle of linear combination in phasor space,^14^ the linear combination of two fluorescent species produces phasor signals that fall on the line drawn between the signals of the two pure species. The relative position along the line represents the proportion of the two pure species in the mixture. This claim is further substantiated as our experimental data, and calibration showed a linear phasor distribution commonly seen in a two-component system rather than a curved FRET trajectory (Figure S4L).

ISG pH is most appropriately quantified with the %Protonation metric derived from in cellulo calibration. Accurate in cellulo 0 and 100% protonation phasor positions are required for robust %Protonation calculation. For the 100% protonation state, using excessive acidity to quench all acceptors will also quench the donor, resulting in an artificially shorter lifetime. Instead, we created a construct with only mTurquoise2 attached to NPY expressed within ISGs and obtained a clean donor-only phasor position incorporating the cell’s autofluorescence (Figure S4I). Removing the acceptors eliminates FRET and yields the same lifetime as the sensors’ theoretical 100% protonation state. By expressing mTurquoise2 in the ISGs, we also verified that ISG pH does not affect mTurquoise2 fluorescence. For the 0% protonation state, we expressed sensors in the mitochondrial lumen (pH 8) to avoid disruption from the permeabilization buffer. Sensors showed high fluorescence colocalization with MitoTracker Red (M7525, Thermo Fisher Scientific), a membrane potential stain for mitochondria (Figure S6). The sensors showed ubiquitous short lifetimes with a small variation. This variation is likely due to small pH variations within mitochondria. We define the 0 and 100% protonation phasor positions by selecting the modes of the respective phasor histogram as we assume that most of the pixels truly represent the sensor behavior in these conditions. The in cellulo calibration phasor aligned well with phasor distributions collected from experiments measuring ISG pH. This calibration also displayed an expected sigmoidal titration curve with a p*K*_a_ around 5.7 (Figure 2D).

### Quantifying Measurement Uncertainty in Live Cells

The ultimate test for these sensors is to precisely measure the pH of each individual ISG within a live cell. We first determined the FLIM photon count needed for a meaningful pH reading. We quantify the pH of an ISG by analyzing pixels in a segmented region of interest (ROI). The uncertainty for this measurement, displayed on the phasor histogram, is the spread of the pixels. This ROI uncertainty decreases as more photons are collected per pixel, making the readings from each pixel more reliable. To determine the photon counts required to generate a reasonable phasor signal from a single ISG, we imaged a single ISG at the same pixel density and dwell time as in actual experiments and controlled the photon gain by the number of scans. In a three-slice *z*-stack of the ISGs (∼1 μm), the phasor distribution from the ROI covering a single ISG displayed a clear mode when the median photon counts of the whole image reached around 60 to 80 per pixel (Figure S7). Although sensor expression differs among cells, this simple criterion allows us to parametrically adjust imaging conditions and select target cells.

**Figure 3:**
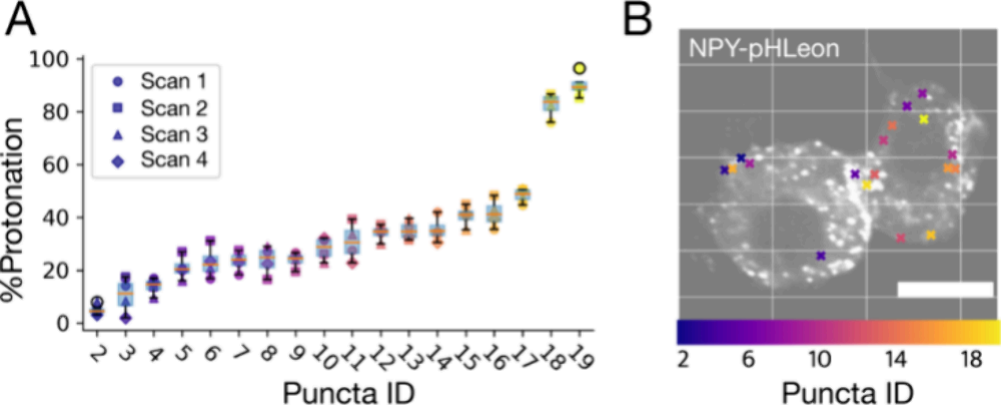
Sensors can discern individual ISG pH in live cell imaging. (A) The error in measurement and segmentation is negligible in ISG pH measurement. Scatterplot of randomly selected ISGs repeatedly measured for %Protonation (*y*-axis). 640 nm thin *z*-slices are imaged four times and then segmented for analysis. Fast-moving and incompletely segmented ISGs are not included in this analysis. (B) Two cells are shown with the localization of specific ISGs selected in (A). Puncta are individual ISGs. The color bar and the data point color in (A) represent the referenced puncta ID. Scale bar = 5 μm.

Next, we determined the uncertainty of measuring ISGs in a living cell with automatic segmentation via Imaris. (Figure S2) This step quantifies the measurement uncertainty associated with the instrument, segmentation, ISG mobility, and laser exposure. We imaged a small *z*-section of a cell spanning the entire volume of most ISGs four times. Each image was subjected to the Imaris automatic segmentation algorithm to identify ISGs. We compared consecutive measurements of relatively stationary ISGs and determined the standard deviations as the measurement uncertainty (Figure 3). The mean uncertainty for repeating ISG pH measurement is ±0.04 pH units. These results demonstrate that a single ISG pH measurement is robust in the experiments.

### ISG pH Landscape in Single Cells

**Figure 4:**
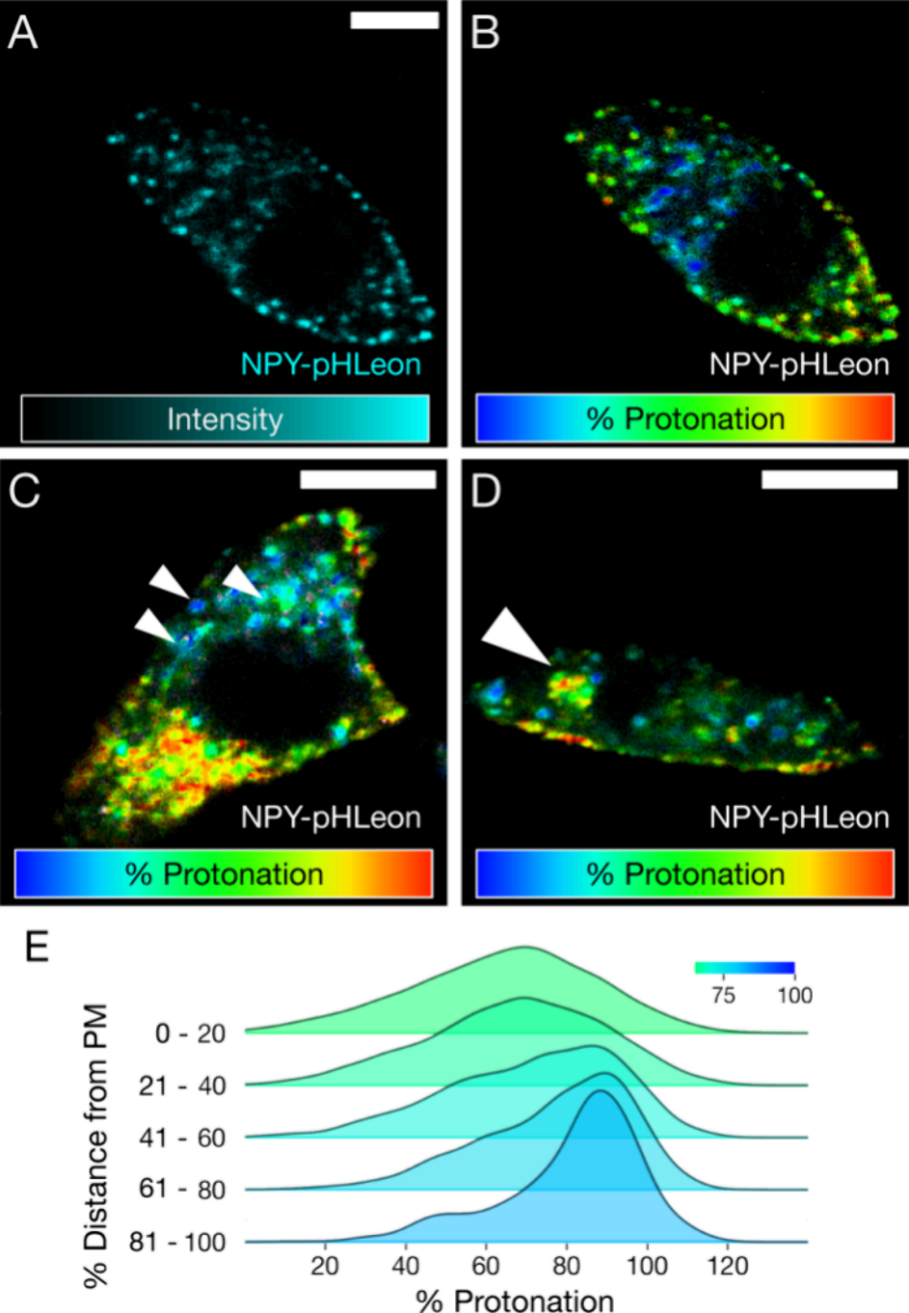
Identification of ISG subpopulations based on pH and location within a single INS-1E cell. (A) Intensity image of a single INS-1E cell expressing NPY-pHLeon imaged in the mTurquoise2 channel. (B) %Protonation measurement is independent of intensity value. Image in (A) overlaid with phasor pseudocolor by the %Protonation value of each pixel. (C) Small triangles point to the acidic multigranular and mobile structures of ISGs located in the cell. (D) Arrowhead highlights a large cluster of less acidic ISGs resembling caged ISGs. (E) Smoothed histogram plots of ISG %Protonation segregated by their relative distance to the plasma membrane. The color of each plot indicates the mean %Protonation of the group. Scale bars = 5 μm.

We next analyzed the entire pH profile of ISGs in INS-1E cells. When evaluating the entire cell, most of the fluorescence is localized into puncta with sizes around 350 nm distributed in both the periphery and interior of the cell, which is consistent with NPY-tagged cells^24^ (Figure 4A). It is worth noting that the observed size for ISGs (∼350 nm) is slightly larger than what has been reported in electron microscopy and soft X-ray tomography studies due to the diffraction limit of light microscopy (Figure 4A).^24−26^ We observed heterogeneity in the ISG pH profiles from cell to cell, which was expected. This variability may be attributed to factors such as the cell cycle, proximity of nearby cells, and cell health. To partially mitigate this variation, we selected a subset of healthy cells based on their morphology, expected size, and distributions of ISGs for further analysis. For example, we did not choose cells that were obviously undergoing stress and had large autophagosomes or cellular defects.

Within this group of healthy cells (*n* = 36), the mean pH value for all ISGs is 5.0 with the median 50% in the range of 5.7–4.8, consistent with previous findings.^27^ With unprecedented resolution and sensitivity, we observed a wide range of pH in all cells and a subset of cells displaying a bimodal distribution of ISG pH. This result clearly highlights the heterogeneity of the chemical environment within ISGs and reflects subpopulations of ISGs (Figure 4). The peripheral ISGs exhibit a more diverse range of pH (median 50% IQR: 5.8–4.8) while the ISGs in the interior lean heavily toward highly acidic (median 50% IQR: 5.0–4.2) (Figure 4E).

It is commonly known that ISGs acidify as they mature.^3^ However, we have limited knowledge of the relationship between ISGs’ spatial organization and maturation.^26,28^ Given that we consistently observe diverse and less acidic ISGs in the cell periphery, it is possible that peripheral ISGs contain multiple populations important for regulating secretion and maturation. Whether pH acts as a signal for recycling or association with exocytosis machinery (SNARE complex) is unknown. Evidence suggests that ISG maturity does not solely depend on association with the plasma membrane since dense core ISGs can appear in both the cell periphery and interior.^25^ In a similar study incorporating a timer protein into ISGs, young (<5 h) ISGs localized predominantly in the periphery, while perinuclear ISGs were usually older.^29,30^ Further investigation is required to explore the relationship between ISG age, maturity, and pH.

A notable finding among the “docked” ISGs near the plasma membrane are instances of ISGs with a pH of 7.4 or higher, while the mean for the region is 5.4 (Figure S8). We believe these are secreting ISGs, given their comparable pH to the extracellular space and proximity to the plasma membrane. The exocytosis behavior of ISGs has been extensively studied with other faster imaging modalities; therefore, it is not elaborated in this study.^19,31^ Yet, further experiments with our pH sensors will provide a new perspective on the timing and location of exocytosis in the whole-cell context.

In the cell’s interior, we capture a combination of moderately and highly acidic ISGs. This range likely represents newly synthesized, maturing, aging, and recycling ISGs. Within the somewhat chaotic cell interior, we often observe multigranular bodies. These clusters of ISGs can reach diameters of 1 μm and are often irregular shapes. Clusters of ISGs have been described before as groupings of ISGs with high insulin content that are potential sites of maturation or a group of ISGs trafficking to the membrane together.^25^ Interestingly, we observe clusters with different pH and morphology, which is consistent with the idea that there are multiple functions for multigranular bodies. We observe that clusters have a pH reaching 6.6 (Figure 4D), which we believe represents caged ISGs.^32^ Caged ISGs are a subpopulation of ISGs stored for future secretion.^33^ We also observed some clusters with a highly acidic pH that reminisces multigranular bodies as precursors for lysosomal degradation (Figure 4C).^8,34^ These clusters appear perinuclear with a slightly larger diameter (∼500 nm) than distinct peripheral ISGs and are highly acidic (pH ∼ 4.4). These acidic granules also exhibit higher mobility in the cell, as demonstrated in the single-frame time-lapse images we acquired (Video S1). Segregating ISG populations by their motion or biochemical characteristics is a widely adopted concept in the field.^7−9,35,36^ Our results provided crucial chemical information about these superstructures and demonstrated the sensitivity of our sensors to capture these details.

### ISG pH Profile during Stimulation in Cells

Previous studies have shown conflicting results on ISG pH during specific stimulation conditions.^37,38^ Thus, we use pHLeon to explore the effect of exocytotic signals on ISG pH to explore these phenomena with higher sensitivity than previously possible.

When monitoring just one ISG over time, we risk missing important details in the broader cellular context that are pivotal for grasping the complete biological picture. Thus, we chose to monitor all ISGs’ pH within a cell at coarser time points during stimulation to learn the influence of stimuli on populations of ISGs based on their cellular location and pH. To determine the baseline variation in the ISG pH profile, we monitored cells with no stimuli. An anticipated small variation in the ISG pH profile is observed, primarily resulting from basal secretion, and natural variation. A pairwise Kolmogorov–Smirnov test across the time points showed no significance between the ISG pH profile at the time points investigated. Thus, we see no change in pH over time under baseline conditions (Figure S9).

We compared three stimulation mechanisms: glucose (metabolic), KCl (membrane depolarization), and exendin-4 (Ex-4) (glucagon-like peptide 1 receptor signaling). Glucose stimulation leads to ISG maturation and secretion.^25^ KCl produces secretion by membrane depolarization, thereby activating voltage-dependent calcium channels, and does not affect ISG maturation.^25^ Ex-4 enhances ISG maturation^25^ but does not cause secretion without costimulation with glucose. Here, we use Ex-4 to validate that ISG maturation will correspond to an ISG acidification. Without the influence from secretion, the measured ISG pH profile in Ex-4-stimulated cells should accurately reflect the acidification process. These conditions provide an opportunity to examine the ISG population pH profile during secretion and maturation. We imaged the same cells before and after stimulation for 40 min. To analyze the results, we compare the ISG pH profiles from before stimulation, 1 to 15 min, and 16 to 40 min after stimulation. This was done to capture two phases of insulin secretion: the acute response from 1 to 15 min after stimulation, and the prolonged response from 16 to 40 min after stimulation.^39,40^

In conditions stimulating secretion (glucose and KCl), the most significant changes in the pH profile come from the peripheral ISGs compared to the whole cell (Figure S10). This result is expected as this cell region will reflect changes due to the secretion of “docked” ISGs and the recruitment of interior reserve pool ISGs to the plasma membrane.^41^ A detailed description for how we define a peripheral vs internal ISG is shown in Figure S11.

**Figure 5:**
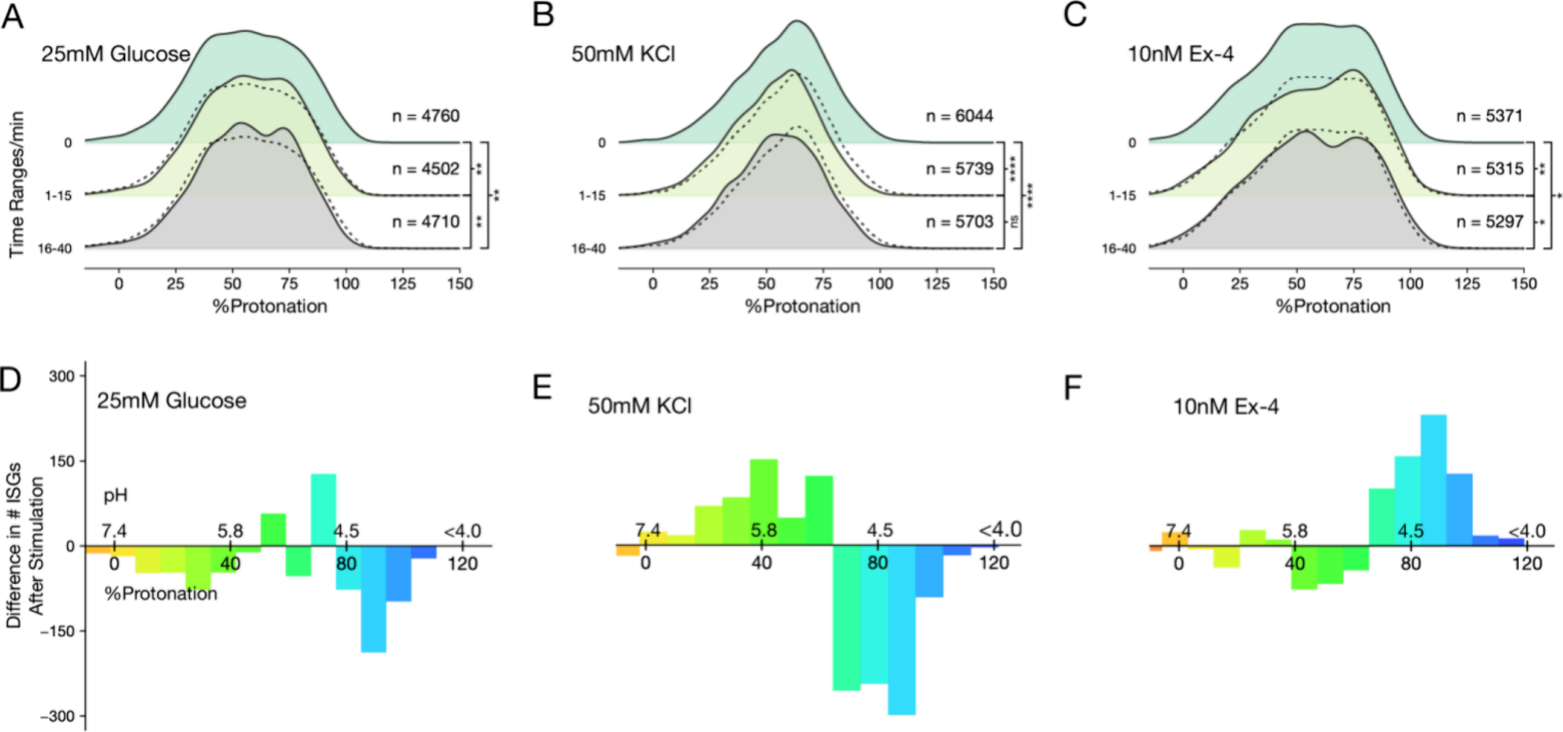
Different insulinotropic stimuli have unique impacts on the pH of ISGs at the cellular periphery: (A–C) The peripheral ISG pH profile is plotted in smoothed normalized histograms showing the full pH range at 0, 1–15 min post stimulation, and 16–40 min post stimulation. The number of ISGs (*n*) for each time point is indicated. Secretion can be noted by a decrease in the number of ISGs over time. Dotted lines represent the basal profile before stimulation. Peripheral ISGs are defined by having a normalized distance to the plasma membrane of less than 0.05 (KS test results: ns: *p* > 0.05, *0.05 < *p* < 0.01, **0.01 < *p* < 0.001, ***0.001 < *p* < 0.0001, number of cells for each condition: Ex-4:21; glucose: 19; KCl: 22). (D–F) ISG pH profile change between 0 (before stimulation) and 1–15 min (after stimulation). Data are binned by %Protonation. Color reflects the same scale of %Protonation used in Figure 4. (A, D) Glucose triggers secretion of more acidic ISGs. (B, E) KCl triggers more prominent secretion with a broader pH range. (C, F) Ex-4 triggers acidification of peripheral ISGs and does not lead to secretion.

In cells treated with 25 mM glucose, we see a reduction of acidic peripheral ISGs (pH < 5.2) within the first 15 min of stimulation (Figure 5D). Cells treated with 50 mM KCl showed a more prominent reduction of ISGs with a broader pH range than glucose stimulation (pH < 5.5) (Figure 5E). This result indicates that the initial secretion of ISGs under glucose is selective toward a population of acidic ISGs in the periphery, while KCl mobilizes a larger portion of the peripheral ISGs with a broader pH range. The ISG number and pH profile changes after 16 min are diminished in these two conditions. This apparent equilibrium at the periphery suggests that ISGs from an internal pool move to the periphery to sustain secretion. The less acidic vesicle population also showed a distinguishable difference between the two secretion conditions. In cells treated with 25 mM glucose, we observed a slight increase in vesicles within the pH range of 5.4–5.2, likely due to the acidification of a less acidic vesicle pool.^25^ On the contrary, this less acidic (pH > 5.5) pool of ISGs increased in the cells treated with 50 mM KCl. This could be caused due to the transient secretion and merging of ISGs with the plasma membrane reducing their pH. This effect is less noticeable in glucose-treated cells because a large pool of vesicles is being acidified outweighing the effect of secretion in alkalizing ISGs.

We tested the nonsecretory stimuli, Ex-4, to directly investigate maturation as opposed to secretion. As expected, cells treated with 10 nM Ex-4 showed no reduction in peripheral ISG count in the first 15 min of stimulation. Significant changes in the peripheral ISG pH profile with Ex-4 include a reduction of ISG around pH 5.7 and enrichment of ISGs around pH 4.5 (Figure 5F). Examining the interior ISGs in a similar manner reveals similar yet less pronounced results (Figure S10). Taken together with the fact that no secretion is observed, it is highly likely that ISGs undergo acidification when treated with Ex-4, aligning with ISG density increase and consistent with the hypothesis that Ex-4 enhances the maturation of ISGs.^25,42^ A notable observation is that ISG numbers are reduced at later time points as we do not anticipate Ex-4 to trigger any ISG release. We suspect that this change in count is due to artifacts such as basal secretion and photobleaching. This is further validated with a similar ISG reduction observed in untreated cells imaged under the same conditions (Figure S9).

## Conclusions

We characterized two genetically encoded FLIM-FRET pH sensors. We demonstrated a pipeline with fast FLIM imaging and phasor analysis that greatly enhanced the precision of these two sensors in live cell imaging with a limited photon budget. We applied these tools to determine pH dynamics within INS-1E cells under different conditions. Our method resolves a single ISG pH and its spatial localization. Our results establish that different stimulation mechanisms impact ISG secretion differently and validate that Ex-4 enhances ISG maturation by altering pH. In the future, a more in-depth analysis of ISG spatial organization, such as contact with other organelles,^42^ would yield additional insights into the cellular mechanisms regulating ISG maturation and secretion. Future experiments using primary beta cells stably expressing our sensors will produce a more accurate model for studying the effect of disease. Lastly, technological advances in imaging that enable single ISG tracking in a whole cell would allow direct investigation of ISG turnover, trafficking, and recycling. A lattice light-sheet microscope with a FLIM camera is a viable candidate for this imaging.

## Supporting Information Available

Rapid movements of ISGs in INS1e cells expressing NPY-pHLeon (MP4)
Experimental details, materials, and methods, including control experimental results (PDF)

## Abbreviations

ISG: insulin secretory granule
FRET: Förster resonance energy transfer
FLIM: fluorescence lifetime imaging microscopy
TCSPC: time-correlated single photon counting
Ex-4: exendin-4 (aka: exenatide)
NPY: neuropeptide Y
FOV: field of view
PM: plasma membrane
IQR: interquartile range
