# Supplementary material for: Decoding Insulin Secretory Granule Maturation Using Genetically Encoded pH Sensors
Source: ACS Sens. 2024 Nov 6;9(11):6032–9. doi: 10.1021/acssensors.4c01885 (PMC11590099; doi:10.1021/acssensors.4c01885)
Supplement: Supplementary file 2 — se4c01885_si_002.pdf [file se4c01885_si_002.pdf]

Supporting information for

Decoding Insulin Secretory Granule Maturation Using Genetically Encoded pH Sensors

Wen Lin[1], Kaylee Tseng[1], Scott E. Fraser[2], Jason Junge[2], Kate L. White[1*]

[1]Department of Chemistry, Bridge Institute, USC Michelson Center for Convergent Bioscience, University of Southern California, Los Angeles, CA 90089, USA.

[2]Department of Biological Sciences, Bridge Institute, USC Michelson Center for Convergent Bioscience, Translational Imaging Center, University of Southern California, 1002 Childs Way, Los Angeles, CA 90089, USA.

*Corresponding Author: Kate L White

Email: katewhit@usc.edu

SUPPLEMENTARY FIGURES

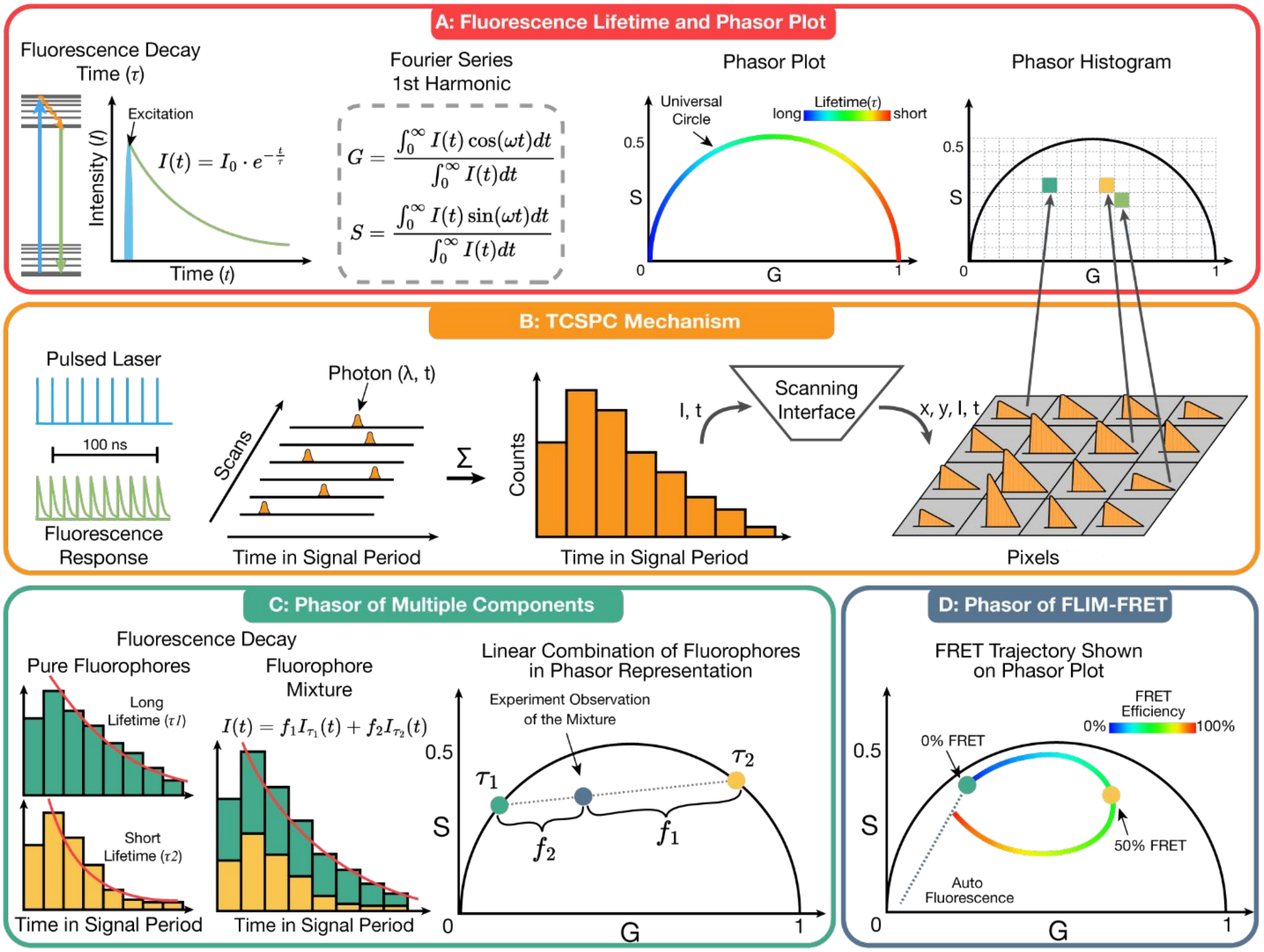

Figure S1. General principles of FLIM imaging and phasor analysis used in this study. (A) Fluorescence lifetime (tau) of a fluorophore is an intrinsic character of the molecule and can be empirically measured through the fluorescence decay function. Phasor analysis, applied in FLIM, uses Fourier transform to convert time-resolved fluorescence decay data into a frequency domain representation, simplifying the interpretation of complex decay dynamics. The universal circle on the phasor plot denotes single component decays with their lifetimes exactly mapped onto the semi-circle. (B) Practical collection of the decay function is achieved with TCSPC instruments. The instrument uses pulsed laser to periodically excite the fluorophores and records the time of flight (TOF) of emitting photons. Compiling the data from multiple scans of the same focal volume generates a histogram that is a close estimate of the decay curve. This process is repeated through a point scanning mechanism to generate phasor values for each pixel which are then plotted onto a phasor histogram, showing the lifetime landscape of an image. (C) Phasor analysis provides an intuitive method to analyze complex decays caused by multiple components. In an experimental system like our pH sensors, more than one fluorophore species is present in a focal volume and their ratio carries relevant information. Phasor analysis uses the illustrated graphical extrapolation to determine their fractions in the focal volume. (D) Phasor analysis can be applied to accurately determine the FRET efficiency of a FRET pair. The FRET trajectory denotes the lifetime characteristic at different FRET efficiencies under the influence of environmental factors. As our sensors are used in cellular context, using FRET trajectory to calculate the FRET efficiency is more accurate than calculating with empirical lifetime values.

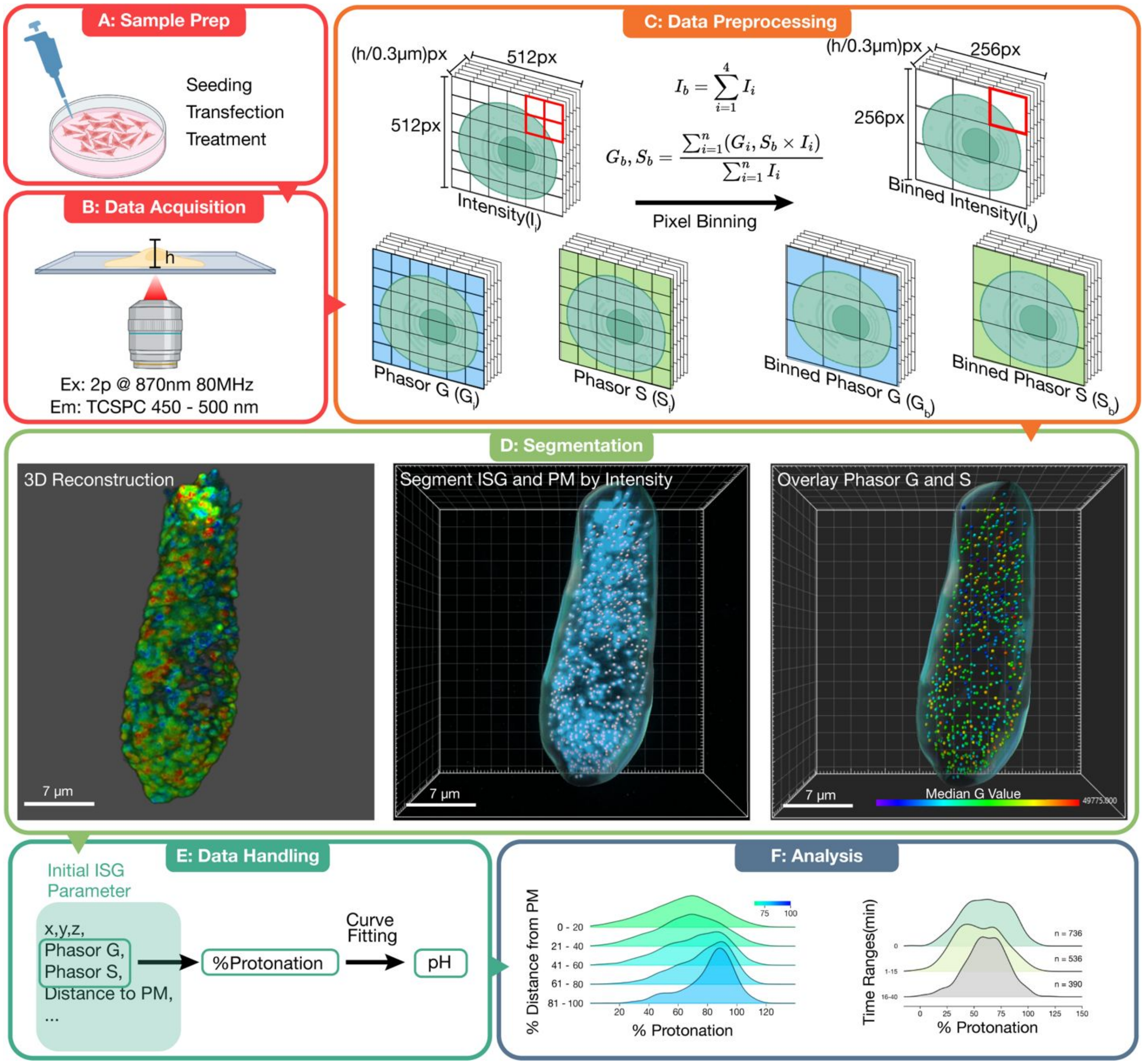

Figure S1. Workflow schematic of data acquisition and analysis. (A) INS-1E cells are grown and treated on imaging-compatible chambered slides as single layer adherent cell culture. (B) Adherent cells are imaged on a bidirectional point-scanning confocal microscope equipped with TCSPC FLIM. The entire thickness of the cell is imaged in series of z-stacks. (C) The FOV is adjusted to contain a single cell and imaged at 512x512 pixels with z-step set at 0.3 µm. Intensity, phasor G, and Phasor S are stored in separate images. The raw images are binned laterally at 2x2, where intensity is the simple summation, and phasor values are weighed means against intensity. The results are three 256x256 images. (D) The images are reconstructed in Imaris10 to generate the 3D cell image. It is then segmented with Imaris particle segmentation algorithm using the intensity channel. The segmented particle mask is then applied to the phasor images and computes the phasor G and S statistics for each segmented punctum. (E) Data generated from segmentation are exported and handled by custom python script. G and S values are used to calculate %Protonation and pH of the puncta. (F) ISG pH profile are subject to analysis with respect to cellular location and stimulation conditions.

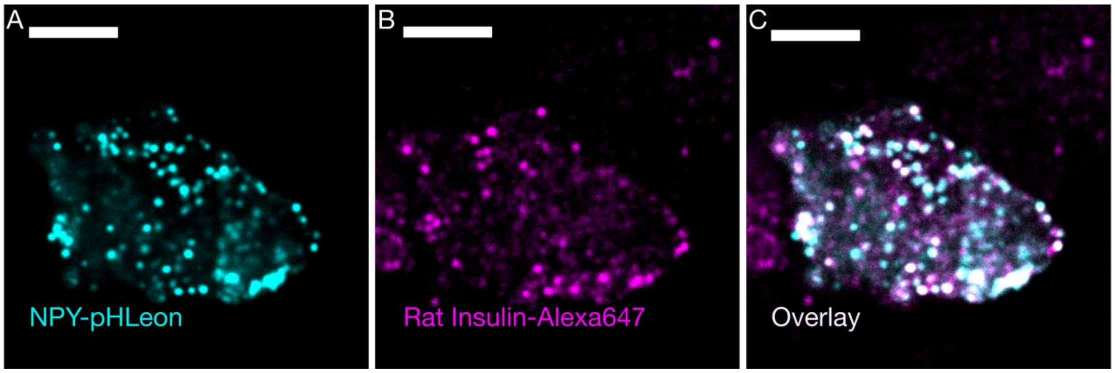

Figure S3: Colocalization of NPY-pHLeon and insulin in INS1e cells. (A) A 3D maximum projection image of an INS1e cell expressing NPY-pHLeon, captured using the mNeonGreen channel. (B) The same field of view as in (A), captured using the Alexa647 channel, where insulin is stained using an anti-insulin antibody. (C) The overlay of images (A) and (B), demonstrating the colocalization of NPY-pHLeon and insulin. The scale bar represents 5 µm. The detailed method for this staining can be found in the supplementary notes of this document.

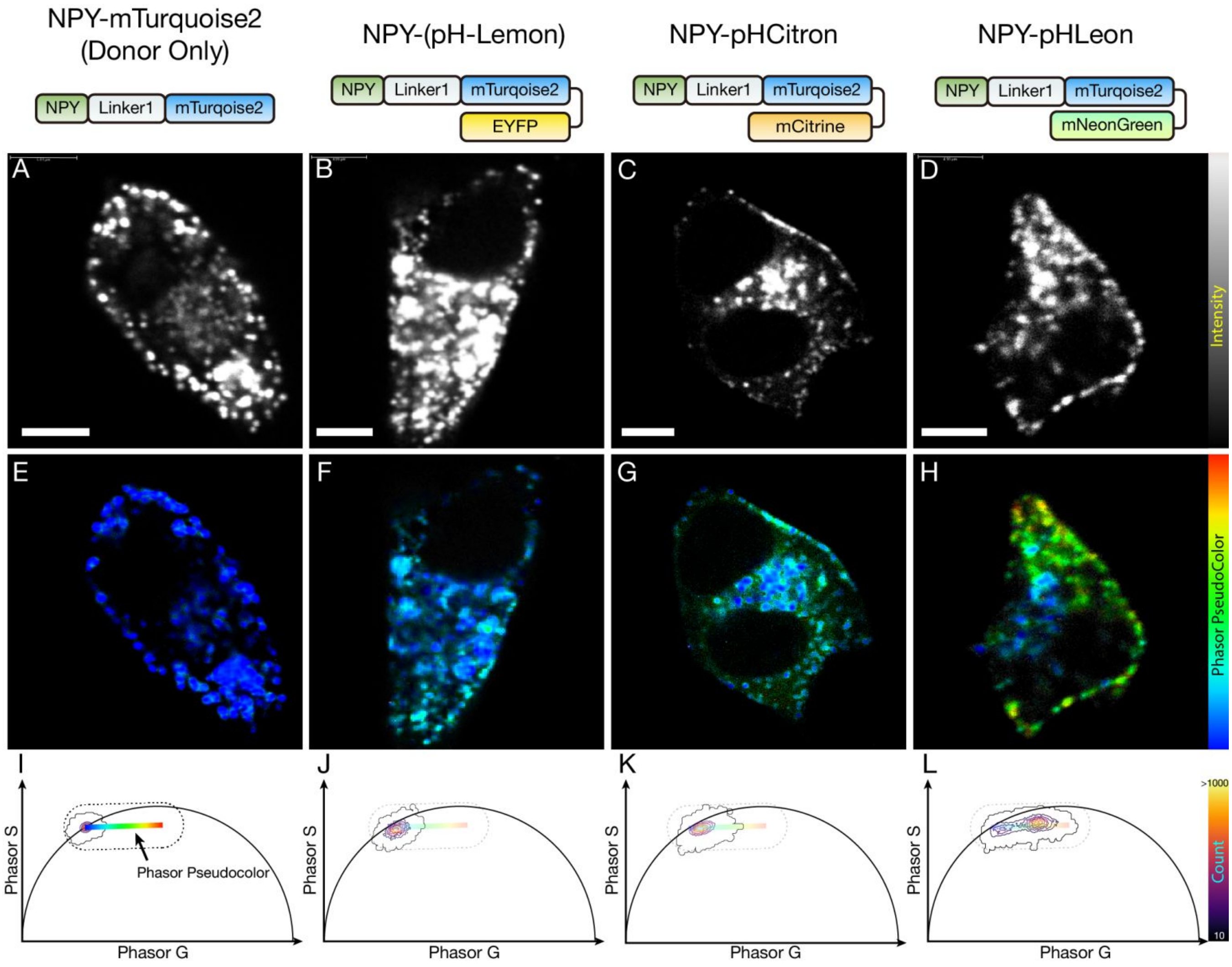

Figure S4. Side-by-side comparison of INS-1E cells expressing pH sensors in ISGs. Top row: Intensity images of INS-1E cells expressing NPY linked to named pH sensor. Middle row: Phasor pseudocolor applied to images in the top row. The color scheme shown in (I) is the same as Figure 4. Bottom row: phasor histogram of middle row images overlayed with the color scale which uses dynamic range of pHLeon as reference to fairly compare the three sensors. (A)(E)(I) The NPY-mTurquoise2 (Donor only) construct showed expected incorporation to ISG. The ubiquitous blue phasor pseudocolor shows that heterogenous ISG pH does not affect the donor's fluorescence lifetime or phasor distribution. (B)(F)(J) pH-Lemon expressed in ISG showed correct incorporation with puncta pattern similar to the donor-only construct. ISG lifetime on phasor plot showed expected lifetime variation in ISGs with representing heterogenous ISG pH. (C)(G)(K) Cells expressing NPY-pHCitron also shows similar puncta pattern as the donor only construct but displayed a wider range of ISG lifetime than pH-lemon as seen on the phasor histogram showed pixels extending into shorter lifetime region on the phasor plot. Since the two constructs share similar native FRET efficiency, we attribute this improvement to mCitrine's lower than EYFP pKa which sensitized the measurement in the pH range of ISGs. (D)(H)(L) The NPY-pHLeon construct also displays the expected punta patterns. The higher native FRET ratio of pHLeon showed a greater ISG lifetime dynamic range extension on the phasor plot, also indicated by the more diverse color shown in the image. Scalebars = 5μm.

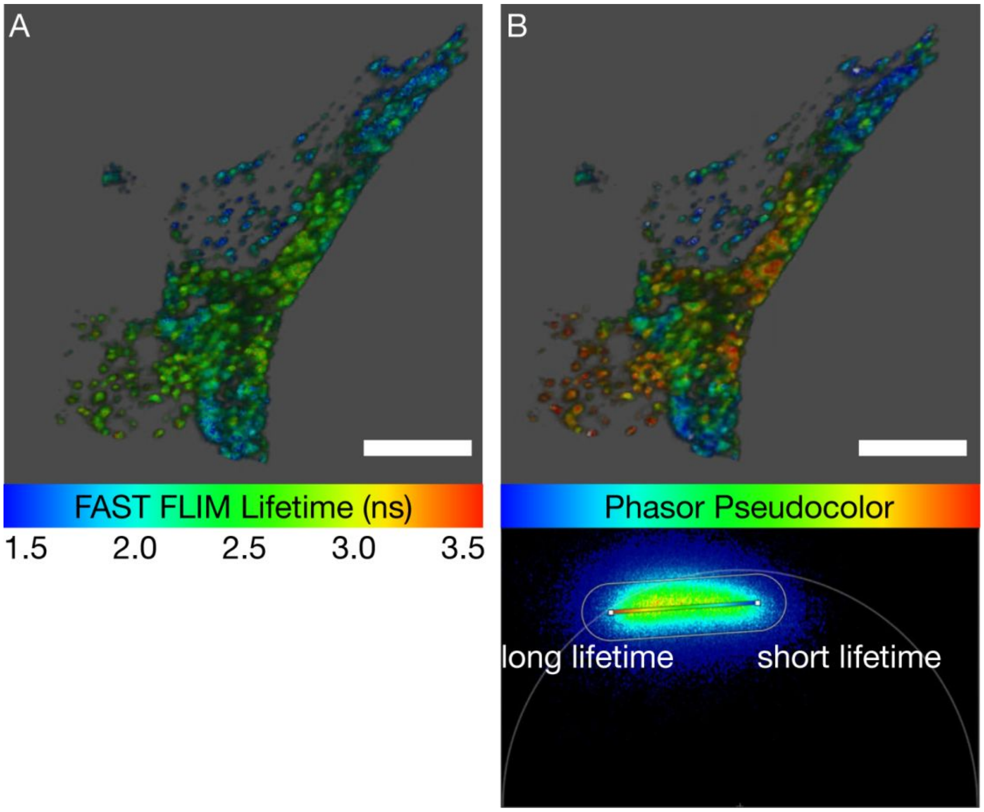

Figure S5. Color code based on phasor analysis showed better separation of lifetime-distinct puncta in live cell images: (A) Fast-FLIM image of a cell z-stack rendered in reconstructed 3D model with each voxel color-coded according to their lifetime. (B) The same image in (A) pseudo-colored with phasor positions is shown in the phasor plot below. Scalebars = 5μm.

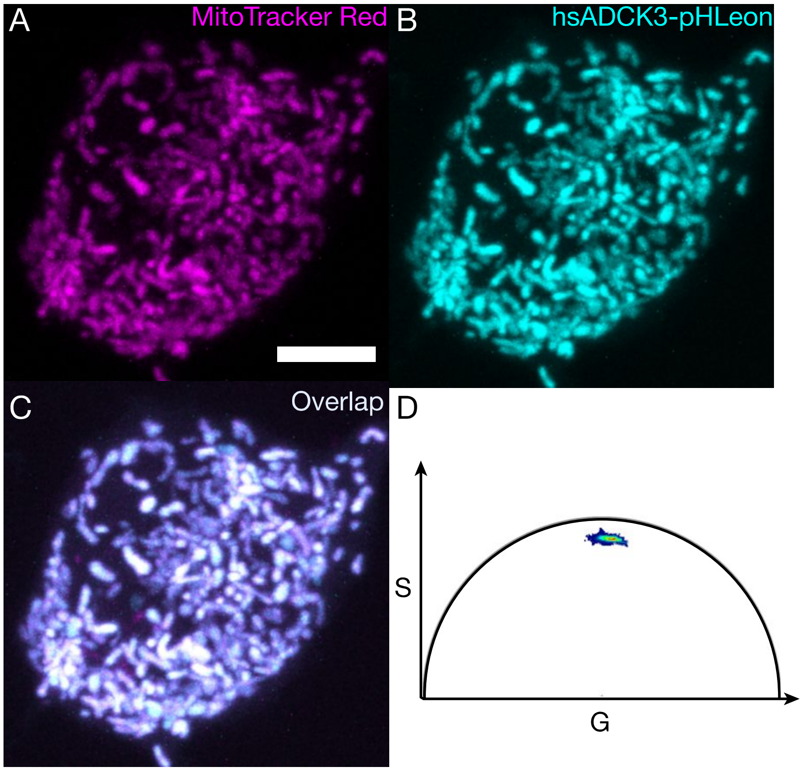

Figure S6. Sensors expressed in alkaline mitochondria lumen shows the max FRET phasor position of the sensor. (A) Max 3D projection of a live cell stained with MitoTracker Red. Scalebar = 5μm. (B) The same cell in (A) expressing mitochondrial pHLeon construct imaged at the acceptor channel, as donor channel is dim due to high FRET ratio. (C) The overlap of the two channels. (D) Phasor histogram of image (B) in donor channel with 40 repetition scans and processed in the same way as other experimental data.

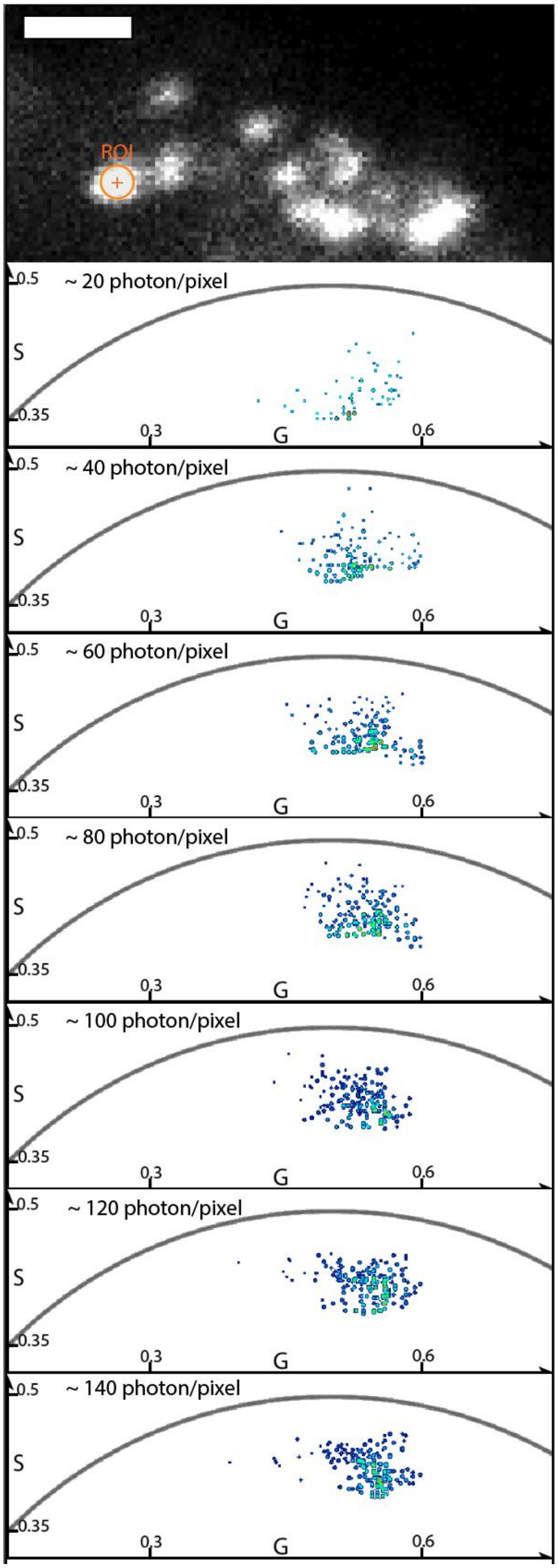

Figure S7. Phasor measurement uncertainty decreases as more photons are collected. Phasor histograms of the same ROI circling a single ISG subjected to repetitive imaging scans with similar pixel density as other cellular experiments. We observed that a consistent mode emerges as the pixel photon count is greater than 60. Scalebars = 1μm.

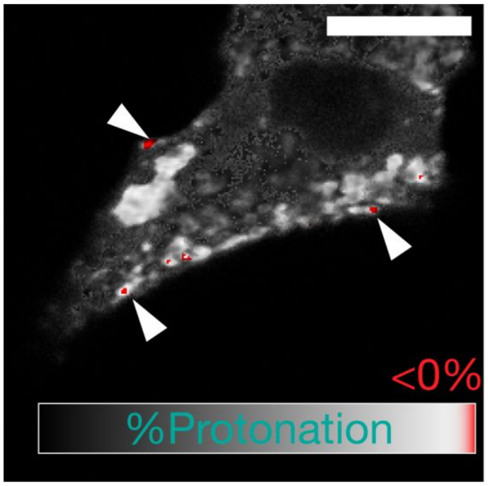

Figure S8. Small arrows point to secreting ISGs along the plasma membrane. Red pixels have %Protonation at 0 or lower indicating their pH is higher than 7.2. Previously mentioned rainbow phasor pseudocolor scheme is switched to grayscale except for the red portion to highlight the sporadic presence of secreting ISGs along the membrane. Scalebars = 5µm.

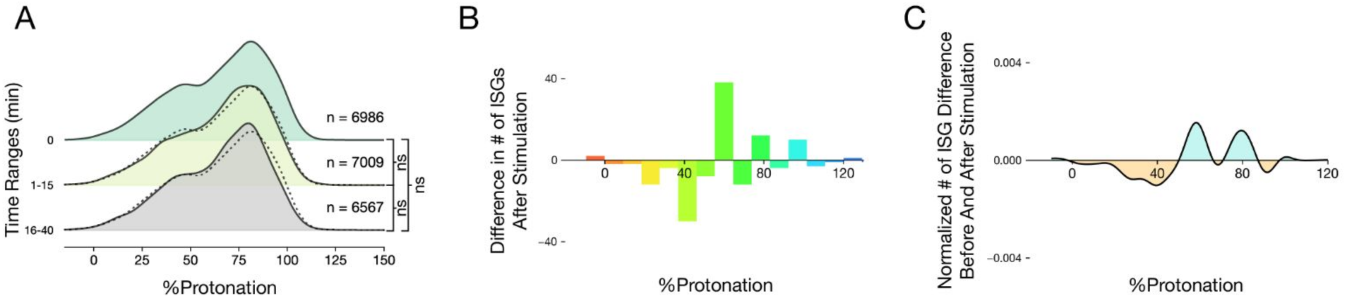

Figure S9. ISG pH profile change overtime with no stimulation: (A) The overall ISG pH profile in cells with no treatment. Data collected in similar manner to stimulation experiments. The different colors represent different timepoints. (B) Difference in counts of ISG pH profile between before simulation and 1-15 after stimulation. (C) Difference histogram shown in (B) normalized against the total number (KS test results: ns: p>0.05, *: 0.05<p<0.01, **: 0.01<p<0.001, ***: 0.001<p<0.0001)

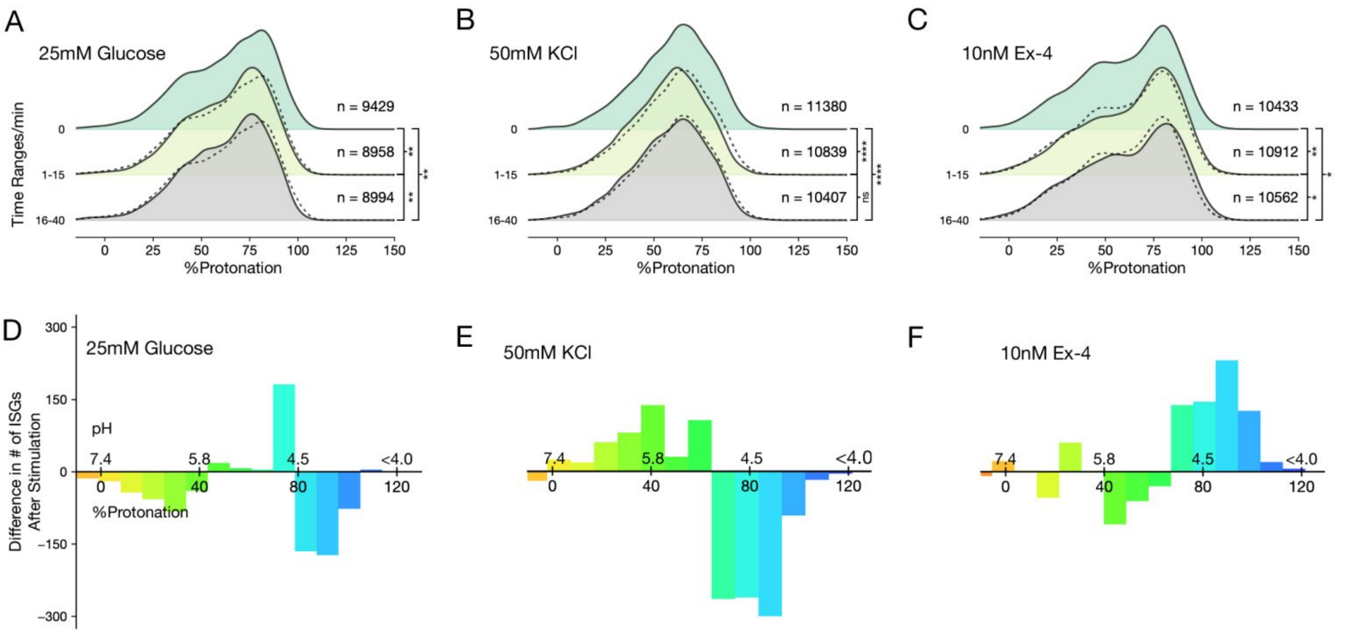

Figure S10. The ISG pH profile of the whole cell change during stimulation plotted similarly as shown in Figure 5, which shows only peripheral ISGs. Trends of ISG pH change during stimulation shown in the whole cell are predominantly contributed by the peripheral ISGs across all conditions. (A)(B)(C) The ISG pH profile of the whole cell is plotted in smoothed normalized histograms showing the full pH range at 0, 1-15 min post stimulation, and 16-40 min post stimulation. The number of ISGs (n) for each timepoint is indicated. Secretion can be noted by a decrease in the number of ISGs over time. Peripheral ISGs are defined by having a normalized distance to the plasma membrane of less than 0.05. (KS test results: ns: p>0.05, *: 0.05<p<0.01, **: 0.01<p<0.001, ***: 0.001<p<0.0001, number of cells for each condition: Ex-4: 21; Glucose: 19; KCl: 22) (D)(E)(F) ISG pH profile change between 0 (before stimulation) and 1–15 min (after stimulation). Data are binned by %Protonation. Color reflects the same scale of %Protonation used in Figure 4. (KS test results: ns: p>0.05, *: 0.05<p<0.01, **: 0.01<p<0.001, ***: 0.001<p<0.0001)

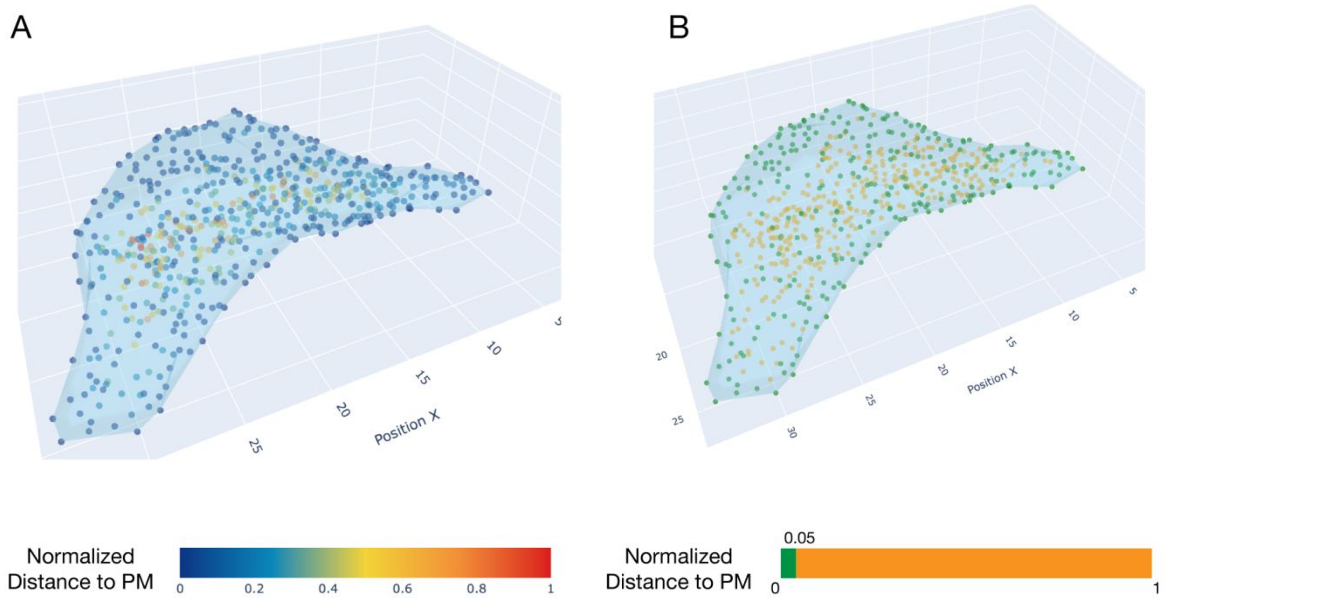

Figure S11. ISG normalized distance to the PM, and the definition of periphery ISGs. (A) Illustration of ISGs in a cell with their calculated normalized distance to PM, presented in segmented ISG coordinates with the boundary mesh of a single cell. Color coded to the calculated normalized distance of each ISG. Smaller value denotes proximity to the boundary, and vice versa. To compensate for variations in cell size, we normalized the ISG distance to the plasma membrane using the Feret radius from the center of mass to the boundary mesh. (B) We define the periphery vesicle to have the normalized distance to PM less than 0.05, illustrated as green in the figure. Unit for position is μm.

SUPPLEMENTARY VIDEO

Video S1: A single focal plane of a INS1e cells expressing NPY-pHLeon, capturing the rapid movements of ISGs. The color-coding corresponds to phasor position, as illustrated in Figure S4. The scale bar represents 5µm.

SUPPLEMENTARY NOTE

MOLECULAR BIOLOGY

pCDNA3.1-NPY-pHCitron were synthesized via GeneScript due to extremely high sequence homology between mTurquoise2 and mCitrine that complicates molecular biology procedures. In the synthesized construct, we included designated restriction sites between each component of the construct to facilitate replacement in future experiments, as illustrated in the sequence schematic (Figure S11). All other plasmids mentioned in the main text are generated by restriction cloning, with DNA inserts made from PCR of templates listed below. All cloning and DNA production is performed with commercial kits (A1223, Promega) and standard procedures. Sequence of product plasmids are confirmed by next generation sequencing.

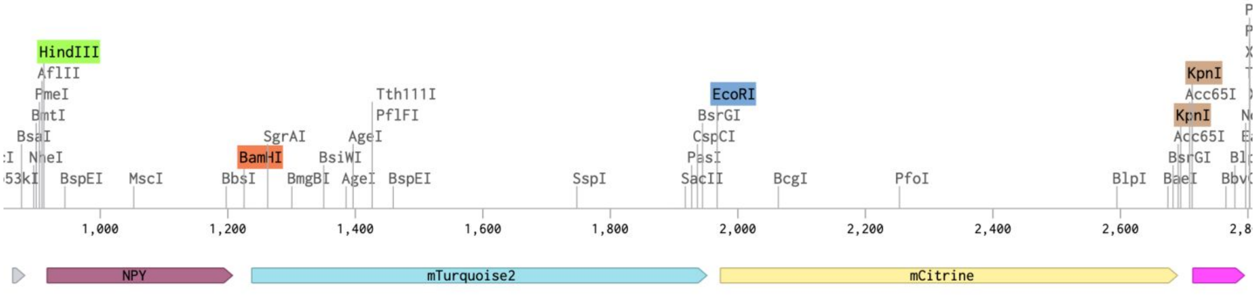

Figure S12. Section of synthesized pCDNA3.1-NPY-pHCitron vector is shown here to highlight the restriction sites used to swap modular components. Designated restriction sites are highlighted in color.

SEQUENCES OF COMMERCIALLY SYNTHESIZED PLASMID

>pCDNA3.1-NPY-pHCitron

GACGGATCGGGAGATCTCCCGATCCCCTATGGTGCACTCTCAGTACAATCTGCTCTGATGCCGCATAGTTAAGCCAGTATCTGCTCCCTGCTTGTGTGTTGGAGGTCGCTGAGTAGTGCGCGAGCAAAATTTAAGCTAC
AACAAGGCAAGGCTTGACCGACAATTGCATGAAGAATCTGCTTAGGGTTAGGCGTTTTGCGCTGCTTCGCGATGTACGGGCCAGATATACGCGTTGACATTGATTATTGACTAGTTATTAATAGTAATCAATTACGGGG
TCATTAGTTCATAGCCCATATATGGAGTTCCGCGTTACATAACTTACGGTAAATGGCCCGCCTGGCTGACCGCCCAACGACCCCCGCCCATTGACGTCAATAATGACGTATGTTCCCATAGTAACGCCAATAGGGACTT
TCCATTGACGTCAATGGGTGGAGTATTTACGGTAAACTGCCCACTTGGCAGTACATCAAGTGTATCATATGCCAAGTACGCCCCCTATTGACGTCAATGACGGTAAATGGCCCGCCTGGCATTATGCCCAGTACATGAC
CTTATGGGACTTTCCTACTTGGCAGTACATCTACGTATTAGTCATCGCTATTACCATGGTGATGCGGTTTTGGCAGTACATCAATGGGCGTGGATAGCGGTTTGACTCACGGGGATTTCCAAGTCTCCACCCCATTGAC
GTCAATGGGAGTTTGTTTTGGCACCAAAATCAACGGGACTTTCCAAAATGTCGTAACAACTCCGCCCCATTGACGCAAATGGGCGGTAGGCGTGTACGGTGGGAGGTCTATATAAGCAGAGCTCTCTGGCTAACTAGAG
AACCCACTGCTTACTGGCTTATCGAAATTAATACGACTCACTATAGGGAGACCCAAGCTGGCTAGCGTTTAAACTTAAGCTTATGCTAGGTAACAAGCGACTGGGGCTGTCCGGACTGACCCTCGCCCTGTCCCTGCTC
GTGTGCCTGGGTGCGCTGGCCGAGGCGTACCCCTCCAAGCCGGACAACCCGGGCGAGGACGCACCAGCGGAGGACATGGCCAGATACTACTCGGCGCTGCGACACTACATCAACCTCATCACCAGGCAGAGATATGGAA
AACGATCCAGCCCAGAGACACTGATTTCAGACCTCTTGATGAGAGAAAGCACAGAAAATGTTCCCAGAACTCGGCTTGAAGACCCTGCAATGTGGACGGTGCCCCGGGCCCGGGATCCCCCAGTAGTTTCAAAAGGAGA
AGAGTTGTTCACCGGCGTGGTTCCGATTCTGGTCGAGTTGGACGGCGACGTGAACGGCCACAAGTTCAGCGTTTCCGGTGAGGGTGAGGGTGACGCGACGTACGGCAAACTGACCCTGAAGTTTATCTGCACCACCGGT
AAGTTACCGGTCCCGTGGCCTACACTGGTAACGACTTTGTCTTGGGGTGTTCAATGCTTCGCCCGTTATCCGGACCACATGAAGCAACATGATTTCTTCAAGAGCGCAATGCCGGAAGGCTACGTTCAAGAGCGCACCA
TCTTTTTCAAAGACGACGGCAACTACAAGACCCGTGCTGAAGTGAAATTCGAGGGCGACACCCTCGTCAACCGCATTGAACTGAAGGGCATTGATTTTAAGGAGGATGGAAACATTCTGGGTCATAAACTGGAATATAA
TTATTTTAGCGATAACGTGTATATCACCGCTGATAAACAGAAAAACGGCATCAAAGCAAATTTTAAGATCCGTCATAATATTGAGGACGGTGGGGTGCAACTGGCGGATCATTACCAGCAGAACACCCCGATTGGTGAC
GGCCCTGTGCTGCTGCCGGATAACCACTACCTGTCCACCCAGAGCAAACTGTCGAAAGATCCGAATGAGAAAAGAGATCACATGGTTCTTTTGGAATTTGTTACAGCCGCGGGCATTACCCTGGGCATGGATGAACTGT
ACAAAGGCGGTGGTGGTTCGGAATTCATGGTGAGCAAGGGCGAGGAGCTGTTCACCGGGGTGGTGCCCATCCTGGTCGAGCTGGACGGCGACGTAAACGGCCACAAGTTCAGCGTGTCCGGCGAGGGCGAGGGCGATGC
CACCTACGGCAAGCTGACCCTGAAGTTCATCTGCACCACCGGCAAGCTGCCCGTGCCCTGGCCCACCCTCGTGACCACCTTCGGCTACGGCCTGATGTGCTTCGCCCGCTACCCCGACCACATGAAGCAGCACGACTTC
TTCAAGTCCGCCATGCCCGAAGGCTACGTCCAGGAGCGCACCATCTTCTTCAAGGACGACGGCAACTACAAGACCCGCGCCGAGGTGAAGTTCGAGGGCGACACCCTGGTGAACCGCATCGAGCTGAAGGGCATCGACT
TCAAGGAGGACGGCAACATCCTGGGGCACAAGCTGGAGTACAACTACAACAGCCACAACGTCTATATCATGGCCGACAAGCAGAAGAACGGCATCAAGGTGAACTTCAAGATCCGCCACAACATCGAGGACGGCAGCGT
GCAGCTCGCCGACCACTACCAGCAGAACACCCCCATCGGCGACGGCCCCGTGCTGCTGCCCGACAACCACTACCTGAGCTACCAGTCCAAGCTGAGCAAAGACCCCAACGAGAAGCGCGATCACATGGTCCTGCTGGAG
TTCGTGACCGCCGCCGGGATCACTCTCGGCATGGACGAGCTGTACAAGGGTACCTAAGAGTCTTAGGGTACCGACTGCAACGCGGCAGGGGCCCTGCGCTTCAGCCTGCCCTCAGTCCTGCTCCTCAGCCTCTTCAGCT
TAGCTTGTCTGTAAGCGGCCGCTCGAGTCTAGAGGGCCCGTTTAAACCCGCTGATCAGCCTCGACTGTGCCTTCTAGTTGCCAGCCATCTGTTGTTTGCCCCTCCCCCGTGCCTTCCTTGACCCTGGAAGGTGCCACTC
CCACTGTCCTTTCCTAATAAAATGAGGAAATTGCATCGCATTGTCTGAGTAGGTGTCATTCTATTCTGGGGGGTGGGGTGGGGCAGGACAGCAAGGGGGAGGATTGGGAAGACAATAGCAGGCATGCTGGGGATGCGGT
GGGCTCTATGGCTTCTGAGGCGGAAAGAACCAGCTGGGGCTCTAGGGGGTATCCCCACGCGCCCTGTAGCGGCGCATTAAGCGCGGCGGGTGTGGTGGTTACGCGCAGCGTGACCGCTACACTTGCCAGCGCCCTAGCG
CCCGCTCCTTTCGCTTTCTTCCCTTCCTTTCTCGCCACGTTCGCCGGCTTTCCCCGTCAAGCTCTAAATCGGGGGCTCCCTTTAGGGTTCCGATTTAGTGCTTTACGGCACCTCGACCCCAAAAAACTTGATTAGGGTG
ATGGTTCACGTAGTGGGCCATCGCCCTGATAGACGGTTTTTCGCCCTTTGACGTTGGAGTCCACGTTCTTTAATAGTGGACTCTTGTTCCAAACTGGAACAACACTCAACCCTATCTCGGTCTATTCTTTTGATTTATA
AGGGATTTTGCCGATTTCGGCCTATTGGTTAAAAAATGAGCTGATTTAACAAAAATTTAACGCGAATTAATTCTGTGGAATGTGTGTCAGTTAGGGTGTGGAAAGTCCCCAGGCTCCCCAGCAGGCAGAAGTATGCAAA
GCATGCATCTCAATTAGTCAGCAACCAGGTGTGGAAAGTCCCCAGGCTCCCCAGCAGGCAGAAGTATGCAAAGCATGCATCTCAATTAGTCAGCAACCATAGTCCCGCCCCTAACTCCGCCCATCCCGCCCCTAACTCC
GCCCAGTTCCGCCCATTCTCCGCCCCATGGCTGACTAATTTTTTTTATTTATGCAGAGGCCGAGGCCGCCTCTGCCTCTGAGCTATTCCAGAAGTAGTGAGGAGGCTTTTTTGGAGGCCTAGGCTTTTGCAAAAAGCTC
CCGGGAGCTTGTATATCCATTTTCGGATCTGATCAAGAGACAGGATGAGGATCGTTTCGCATGATTGAACAAGATGGATTGCACGCAGGTTCTCCGGCCGCTTGGGTGGAGAGGCTATTCGGCTATGACTGGGCACAAC
AGACAATCGGCTGCTCTGATGCCGCCGTGTTCCGGCTGTCAGCGCAGGGGCGCCCGGTTCTTTTTGTCAAGACCGACCTGTCCGGTGCCCTGAATGAACTGCAGGACGAGGCAGCGCGGCTATCGTGGCTGGCCACGAC
GGGCGTTCCTTGCGCAGCTGTGCTCGACGTTGTCACTGAAGCGGGAAGGGACTGGCTGCTATTGGGCGAAGTGCCGGGGCAGGATCTCCTGTCATCTCACCTTGCTCCTGCCGAGAAAGTATCCATCATGGCTGATGCA
ATGCGGCGGCTGCATACGCTTGATCCGGCTACCTGCCCATTCGACCACCAAGCGAAACATCGCATCGAGCGAGCACGTACTCGGATGGAAGCCGGTCTTGTCGATCAGGATGATCTGGACGAAGAGCATCAGGGGCTCG
CGCCAGCCGAACTGTTCGCCAGGCTCAAGGCGCGCATGCCCGACGGCGAGGATCTCGTCGTGACCCATGGCGATGCCTGCTTGCCGAATATCATGGTGGAAAATGGCCGCTTTTCTGGATTCATCGACTGTGGCCGGCT
GGGTGTGGCGGACCGCTATCAGGACATAGCGTTGGCTACCCGTGATATTGCTGAAGAGCTTGGCGGCGAATGGGCTGACCGCTTCCTCGTGCTTTACGGTATCGCCGCTCCCGATTCGCAGCGCATCGCCTTCTATCGC
CTTCTTGACGAGTTCTTCTGAGCGGGACTCTGGGGTTCGAAATGACCGACCAAGCGACGCCCAACCTGCCATCACGAGATTTCGATTCCACCGCCGCCTTCTATGAAAGGTTGGGCTTCGGAATCGTTTTCCGGGACGC
CGGCTGGATGATCCTCCAGCGCGGGGATCTCATGCTGGAGTTCTTCGCCCACCCCAACTTGTTTATTGCAGCTTATAATGGTTACAAATAAAGCAATAGCATCACAAATTTCACAAATAAAGCATTTTTTTCACTGCAT
TCTAGTTGTGGTTTGTCCAAACTCATCAATGTATCTTATCATGTCTGTATACCGTCGACCTCTAGCTAGAGCTTGGCGTAATCATGGTCATAGCTGTTTCCTGTGTGAAATTGTTATCCGCTCACAATTCCACACAACA
TACGAGCCGGAAGCATAAAGTGTAAAGCCTGGGGTGCCTAATGAGTGAGCTAACTCACATTAATTGCGTTGCGCTCACTGCCCGCTTTCCAGTCGGGAAACCTGTCGTGCCAGCTGCATTAATGAATCGGCCAACGCGC
GGGGAGAGGCGGTTTGCGTATTGGGCGCTCTTCCGCTTCCTCGCTCACTGACTCGCTGCGCTCGGTCGTTCGGCTGCGGCGAGCGGTATCAGCTCACTCAAAGGCGGTAATACGGTTATCCACAGAATCAGGGGATAAC
GCAGGAAAGAACATGTGAGCAAAAGGCCAGCAAAAGGCCAGGAACCGTAAAAAGGCCGCGTTGCTGGCGTTTTTCCATAGGCTCCGCCCCCCTGACGAGCATCACAAAAATCGACGCTCAAGTCAGAGGTGGCGAAACC
CGACAGGACTATAAAGATACCAGGCGTTTCCCCCTGGAAGCTCCCTCGTGCGCTCTCCTGTTCCGACCCTGCCGCTTACCGGATACCTGTCCGCCTTTCTCCCTTCGGGAAGCGTGGCGCTTTCTCATAGCTCACGCTG
TAGGTATCTCAGTTCGGTGTAGGTCGTTCGCTCCAAGCTGGGCTGTGTGCACGAACCCCCCGTTCAGCCCGACCGCTGCGCCTTATCCGGTAACTATCGTCTTGAGTCCAACCCGGTAAGACACGACTTATCGCCACTG
GCAGCAGCCACTGGTAACAGGATTAGCAGAGCGAGGTATGTAGGCGGTGCTACAGAGTTCTTGAAGTGGTGGCCTAACTACGGCTACACTAGAAGAACAGTATTTGGTATCTGCGCTCTGCTGAAGCCAGTTACCTTCG
GAAAAAGAGTTGGTAGCTCTTGATCCGGCAAACAAACCACCGCTGGTAGCGGTGGTTTTTTTGTTTGCAAGCAGCAGATTACGCGCAGAAAAAAAGGATCTCAAGAAGATCCTTTGATCTTTTCTACGGGGTCTGACGC
TCAGTGGAACGAAAACTCACGTTAAGGGATTTTGGTCATGAGATTATCAAAAAGGATCTTCACCTAGATCCTTTTAAATTAAAAATGAAGTTTTAAATCAATCTAAAGTATATATGAGTAAACTTGGTCTGACAGTTAC
CAATGCTTAATCAGTGAGGCACCTATCTCAGCGATCTGTCTATTTCGTTCATCCATAGTTGCCTGACTCCCCGTCGTGTAGATAACTACGATACGGGAGGGCTTACCATCTGGCCCCAGTGCTGCAATGATACCGCGAG
ACCCACGCTCACCGGCTCCAGATTTATCAGCAATAAACCAGCCAGCCGGAAGGGCCGAGCGCAGAAGTGGTCCTGCAACTTTATCCGCCTCCATCCAGTCTATTAATTGTTGCCGGGAAGCTAGAGTAAGTAGTTCGCC
AGTTAATAGTTTGCGCAACGTTGTTGCCATTGCTACAGGCATCGTGGTGTCACGCTCGTCGTTTGGTATGGCTTCATTCAGCTCCGGTTCCCAACGATCAAGGCGAGTTACATGATCCCCCATGTTGTGCAAAAAAGCG
GTTAGCTCCTTCGGTCCTCCGATCGTTGTCAGAAGTAAGTTGGCCGCAGTGTTATCACTCATGGTTATGGCAGCACTGCATAATTCTCTTACTGTCATGCCATCCGTAAGATGCTTTTCTGTGACTGGTGAGTACTCAA
CCAAGTCATTCTGAGAATAGTGTATGCGGCGACCGAGTTGCTCTTGCCCGGCGTCAATACGGGATAATACCGCGCCACATAGCAGAACTTTAAAAGTGCTCATCATTGGAAAACGTTCTTCGGGGCGAAAACTCTCAAG
GATCTTACCGCTGTTGAGATCCAGTTCGATGTAACCCACTCGTGCACCCAACTGATCTTCAGCATCTTTTACTTTCACCAGCGTTTCTGGGTGAGCAAAAACAGGAAGGCAAAATGCCGCAAAAAAGGGAATAAGGGCG
ACACGGAAATGTTGAATACTCATACTCTTCCTTTTTCAATATTATTGAAGCATTTATCAGGGTTATTGTCTCATGAGCGGATACATATTTGAATGTATTTAGAAAAATAAACAAATAGGGGTTCCGCGCACATTTCCCC
GAAAAGTGCCACCTGACGTC

Table 1: PCR primers and templates used to create constructs used in this study.

Construct	Target Fragment	Target Sequence or Source Plasmid Addgene ID	Forward Primer	Reverse Primer
**pCDNA3.1-NPY-pHLeon**	mNeonGreen	125139	ATGCTAGAATTCATGGTGAGC AAGGGCGAGGAGGATAAC	TACGATGGTACCTTACTTG TACAGCTCGTCCATGCCC
**pCDNA3.1-hsADCK3-pHLeon**	hsADCK3	ATGGCGGCGATTCTGGGCGATACCATTATGGTGGCGAAAGGCCTGGTGAAACTG ACCCAGGCGGCGGTGGAAACCCATCTGCAGCATCTGGGCATTGGCGGCGAACTG ATTATGGCGGCGCGCGCGCTGCAGAGCACCGCGGTGGAACAGATTGGCATGTTT CTGGGCAAAGTGCAGGGCCAGGATAAACATGAAGAATATTTTGCGGAAAACTTT GGCGGCCCGGAAGGCGAATTTCATTTTAGCGTGCCGCATGCGGCGGGCGCGAGC ACCGATTTTAGCAGCGCGAGCGCGCCGGATCAGAGCGCGCCGCCGAGCCTGGGC CATGCGCATAGCGAAGGCCCGGCGCCGGCGTATGTGGCGAGCGGCCCGTTTCGC GAAGCGGGCTTTCCGGGCCAGGCGAGCAGCCCGCTGGGCCGCGCGAACGGCCGC CTGTTTGCGAACCCGCGCGATAGCTTTAGCGCGATGGGCTTTCAGCGCCGCTTT	N/A Synthesized	N/A Synthesized
**pCDNA3.1-pHLeon**	Remove NPY	-	AGCTGGCTAGCGTTTAAACTT AAGCTTATGCGGGATCCCCCA GTAGTTTCAAAAGGAGAA	TTCTCCTTTTGAAACTACT GGGGGATCCCGCATAAGCT TAAGTTTAAACGCTAGCCA GCT

Table 2: Protein Sequences of Linkers Referenced in Figure 1

Linker	Protein Sequence
**Linker 1**	TVPRARDPPV
**Linker 2**	GGGGSEF

BUFFERS AND MEDIA

Supplemented RPMI 1640 medium: RPMI 1640 medium (11875093, Thermo Fisher Scientific),11 mM glucose, 5% FBS, 100 units/mL penicillin, 100 µg/mL streptomycin, 2 mM glutamine, 10 mM HEPES, 1 mM sodium pyruvate and 50µM β -mercaptoethanol.
KRBH buffer: 135 mM NaCl, 3.6 mM KCl, 5 mM $NaHCO_3$, 0.5 mM NaH_2PO_4, 0.5 mM $MgCl_2$, 1.5 mM $CaCl_2$, 0.1% BSA and 10 mM HEPES, pH 7.4.
pH calibration buffers: combinations of 0.1 M citric acid titrated with 0.2 M NaH_2PO_4 to specific pH in saline.
Blocking buffer: PBS with 1% bovine serum albumin, pH 7.4.
Staining buffer: PBS with 1% bovine serum albumin, and 0.1% sodium azide. pH 7.4.

FIXED CELL STAINING

INS1e cells, grown in chambered slides until reaching 70% confluency, are transfected with Lipofectamine 3000 reagent following the manufacturer's protocol to express NPY-pHLeon. Twenty-four hours post-transfection, the cells are fixed with cold 4% paraformaldehyde in PBS (Thermo Fisher Scientific #J19943.K2) on ice for 30 minutes. After fixation, the cells are washed again and incubated in blocking buffer for one hour at room temperature.

After blocking, the cells are washed three times with PBS and stained with primary antibody Insulin (L6B10) Mouse mAb (Cell Signaling Technology #8138S) at a concentration of 1:400 for 2 hours at room temperature on a rocking platform. The secondary antibody conjugated with Alexa Flour 647 (Thermo Fisher Scientific #A-21236) is added similarly at a concentration of 1:400 and stained for 2 hours. After staining, the cells are washed three times and then imaged in PBS.

DATA ANALYSIS

To improve the signal-to-noise ratio, each lateral slice of the image was binned at 2x2 pixels, resulting in pixels with compounded photon counts (the sum of 4 pixels). The mean of the phasor G and S coordinates were weighted by the photon counts and stored in two separate image channels (Figure S1).
Pixels with a photon count lower than 10 were excluded from further analysis. The processed image was then exported to Imaris10 (Oxford Instruments) for reconstruction and segmentation.
Segmentation of the ISG was performed using the particle segmentation function in Imaris10. The segmented particles were represented as ellipsoids with a diameter of 350 nm and 2x elongation along the z-axis, resembling the confocal system's point spread function. Segmented ISGs are subject to manual thresholding by gating the "mean intensity" and "quality" of the puncta, two internal parameters calculated by Imaris. To determine the ISG distance to the plasma membrane, we used a custom script to compute 3D alpha shape boundaries for each cell image. The parameters were manually adjusted to ensure the boundary mesh aligned with the actual image. The Euclidean distance from each segmented vesicle to the boundary mesh was recorded and normalized against the distance to the center of mass of the boundary mesh, to reduce the influence of cell size variations.
The data of interest was quantified using these segmentations. For each segmented ISG, we acquired total photon counts, mean, and median values of phasor G and S with standard deviation, shortest distance to the segmented cell boundary, and spatial coordinates regarding the image. pH values for the ISG were extrapolated from the G and S values by converting them to %Protonation through linear regression. The pH values were then calculated by fitting them to a calibration curve against the in cellulo calibration standards. The exported ISG data were handled using a custom Python script, available upon request.
